# Associations of Sedentary and Physically-Active Behaviors With Cognitive-Function Decline in Community-Dwelling Older Adults: Compositional Data Analysis From the NEIGE Study

**DOI:** 10.2188/jea.JE20190141

**Published:** 2020-11-05

**Authors:** Shiho Amagasa, Shigeru Inoue, Hiroshi Murayama, Takeo Fujiwara, Hiroyuki Kikuchi, Noritoshi Fukushima, Masaki Machida, Sebastien Chastin, Neville Owen, Yugo Shobugawa

**Affiliations:** 1 Department of Preventive Medicine and Public Health, Tokyo Medical University, Tokyo, Japan; 2 Institute of Gerontology, The University of Tokyo, Tokyo, Japan; 3 Department of Global Health Promotion, Tokyo Medical and Dental University, Tokyo, Japan; 4 School of Health and life Science, Institute of Applied Health Research, Glasgow Caledonian University, Glasgow, United Kingdom; 5 Department of Sport and Movement Science, Ghent University, Ghent, Belgium; 6 Behavioral Epidemiology Laboratory, Baker Heart & Diabetes Institute, Melbourne, Victoria, Australia; 7 Centre for Urban Transitions, Swinburne University of Technology, Melbourne, Victoria, Australia; 8 Division of International Health, Niigata University Graduate School of Medical and Dental Sciences, Niigata, Japan; 9 Department of Active Ageing, Niigata University Graduate School of Medical and Dental Sciences, Niigata, Japan

**Keywords:** accelerometry, aging, exercise, sedentary lifestyle, neurocognitive disorders

## Abstract

**Background:**

Physical activity can help to protect against cognitive decline in older adults. However, little is known about the potential combined relationships of time spent in sedentary behavior (SB), light-intensity physical activity (LPA), and moderate-to-vigorous physical activity (MVPA) with indices of cognitive health. We examined the cross-sectional associations of objectively-determined sedentary and physically-active behaviors with an indicator of cognitive function decline (CFD) in older adults.

**Methods:**

A randomly-recruited sample of 511 Japanese older adults (47% male; aged 65–84 years) wore a tri-axial accelerometer for 7 consecutive days in 2017. Cognitive function was assessed by interviewers using the Japanese version of Mini-Mental State Examination, with a score of ≤23 indicating CFD. Associations of sedentary and physically-active behaviors with CFD were examined using a compositional logistic regression analysis based on isometric log-ratio transformations of time use, adjusting for potential confounders.

**Results:**

Forty one (9.4%) of the participants had an indication of CFD. Activity compositions differed significantly between CFD and normal cognitive function (NCF); the proportion of time spent in MVPA was 39.1% lower, relative to the overall mean composition in those with CFD, and was 5.3% higher in those with NCF. There was a significant beneficial association of having a higher proportion of MVPA relative to other activities with CFD. LPA and SB were not associated with CFD when models were corrected for time spent in all activity behaviors.

**Conclusions:**

Larger relative contribution of MVPA was favorably associated with an indicator of CFD in older adults.

## INTRODUCTION

Dementia is an increasing public health concern worldwide.^1^ A meta-analysis of the global literature on the prevalence of dementia estimated that 35.6 million people lived with dementia across the world in 2010, with numbers expected to almost double every 20 years, to 65.7 million in 2030 and 115.4 million in 2050.^2^ High prevalence of dementia also represents a huge global economic cost.^3^ Effective preventive strategies are urgently needed.

Physical activity can help in preventing the onset of dementia and decline of cognitive function. However, some studies have identified protective effects,^4^^,^^5^ whereas many have shown no apparent benefits.^6^^–^^8^ Sedentary behavior (SB; time spent sitting) may have a deleterious effect on cognitive health.^9^^–^^11^ Longer time spent in SB has been found to be associated with poorer cognitive function in older adults, but this association was attenuated after taking into account moderate-to-vigorous physical activity (MVPA).^11^ Light-intensity physical activity (LPA) has also been examined in this context.^12^^,^^13^ A longitudinal study indicated longer time spent in LPA may prevent cognitive decline, after controlling for time spent in MVPA.^12^ Such studies of the relationships of sedentary and physically-active behaviors with indices of cognitive health have included basic statistical adjustment for time spent in the other behaviors. The combined relationships of time spent in SB and intensity-specific physical activity with cognitive health remain to be examined.

Time is finite during the day, and activity behaviors are not independent. Compositional data analysis (CoDa) allows the examination of co-dependence of time spent in all behaviors arising within a day or part of the day.^14^^,^^15^ For example, if time spent in MVPA increases or decreases, this can influence the time spent in SB and LPA. Findings obtained using basic statistical adjustment for physical activity have differed from those of studies using CoDa.^14^^,^^16^ Conventional statistical models can be misleading, with some effects being over- or under-estimated. To date, no previous study has investigated a role of each activity behavior with indices of cognitive health when time spent in other activities is taken into account.

We examined the associations of objectively-determined SB, LPA, and MVPA with cognitive function in community-dwelling older adults using the CoDa approach. We also explored relationships of bout-length specific MVPA with an index of cognitive function.

## METHODS

### Study sample and data collection

This cross-sectional study was a part of the Neuron to Environmental Impact across Generations (NEIGE) study.^17^ Participants were community-dwelling older adults without long-term care in Tokamachi city, Niigata Prefecture, Japan. Tokamachi is a rural city located in the southernmost region of Niigata Prefecture (area: 590.4 km^2^, population: 54,515, as of February 8, 2018). A total of 1,346 residents (aged 65–84 years) were selected from a resident registry using stratified random sampling. In the fall of 2017, we conducted a questionnaire survey and health examination to 527 participants who agreed to enroll in NEIGE study, and at the same time they were asked to wear an accelerometer. Detailed methods have been reported elsewhere.^17^

The University Ethics Committee (Niigata University and Tokyo Medical University) granted ethics approval. Written informed consent was obtained from all participants.

### Assessment of activity behaviors (independent variable)

Participants were instructed to wear an accelerometer, the Active style Pro HJA-750C (Omron Healthcare, Kyoto, Japan), over the waist on an elasticated belt for 7 consecutive days while awake, except during water-based activities (eg, swimming and hot springs). Active style Pro is a validated accelerometer^18^^–^^20^ that provides data comparable to the devices most commonly used in studies conducted in Western countries.^21^^,^^22^ Its algorithm has been explained in detail elsewhere.^18^^,^^19^ No acceleration signal being detected for longer than 60 consecutive minutes was defined as “non-wear”, and records from participants wearing the accelerometer for at least 10 hours per day were considered valid.^23^ Participants with 4 or more valid wear days were included in the analyses.^24^^,^^25^ We used 60-second epoch data and obtained estimated metabolic equivalents (METs) values using analysis software. METs-based criteria was used to determine each intensity of activities: ≤1.5 METs for SB, 1.6–2.9 METs for LPA, and ≥3.0 METs for MVPA.^26^^,^^27^ MVPA was further classified according to bout length: sporadic MVPA and bouted MVPA.^28^ Bouted MVPA was defined as 10 or more consecutive minutes above the moderate intensity threshold, with allowance for interruptions of 1 or 2 minutes per 10 minutes below the threshold.^25^ Sporadic MVPA was calculating by subtracting bouted MVPA from total MVPA. The analysis included the sub-compositions of activity behaviors that constitute accelerometer wearing time (SB, LPA, and MVPA).

### Assessment of cognitive function (dependent variable)

Cognitive function was assessed by interviewers using the Japanese version of the Mini-Mental State Examination (MMSE-J).^29^^,^^30^ MMSE has been commonly used for screening dementia and mild cognitive impairment (MCI). MMSE-J was used as a total score (range 0–30) and also divided into the following sub-domains: orientation (score 0–10), working memory (score 0–3), attention (score 0–5), delayed memory (score 0–3), and language (score 0–9). Based on previous research, a total score of ≤23 as cognitive function decline (CFD).^29^ This 23/24 cut-off value classified into a normal cognitive impairment/MCI group and an Alzheimer disease (AD) group with 0.86 sensitivity and 0.89 specificity.^29^

### Covariates

Residential area (city side/countryside) was obtained from residential registry of Tokamachi city. Participants reported their age, gender, living arrangement (with others/alone), working status (workers/non-workers), educational attainment (<13 years/≥13 years), smoking (smokers/non-smokers), and alcohol use (yes/no). Medical doctors asked participants to report their past history of stroke (cerebral infarction, cerebral hemorrhage, and subarachnoid hemorrhage) and the use of medication for hypertension, dyslipidemia, and diabetes. Body mass index (BMI) was calculated from height and weight (kg/m^2^) measured using a body composition analyzer MC-780A (TANITA Corporation, Tokyo, Japan).

### Statistical analyses

R version 3.5.2 (R Foundation for Statistical Computing, Vienna, Austria) was used to perform all statistical analyses. We used R package ‘compositions’, ‘robCompositions’, and ‘zCompositions’ for CoDa approach. Statistical significance was set at *P* < 0.05.

The chi-square test, Fisher’s exact test, or *t*-test was performed to compare participant characteristics between those who with CFD and normal cognitive function (NCF). We adopted a CoDa approach, as detailed in previous research.^14^ Variability in the data, in terms of variability of each behavior relative to the variability of other behaviors, was described through a variation matrix.^14^^,^^31^ A log-ratio expectation-maximization algorithm was used to impute zeros in compositional data sets, since zero does not allow for log-ratio transformation.^32^ One participant (0.2%) and 138 (27.0%) participants had no time spent in MVPA and bouted MVPA, respectively. We graphically described the difference of activity behaviors by cognitive status to initially appraise the relative differences between these groups. To support the graphical interpretation, we used multivariate analysis of variance (MANOVA) to test whether the activity compositions significantly differed overall between CFD and NCF.

To investigate associations of activity behaviors with CFD, a compositional multiple logistic regression analysis using isometric log-ratio (ilr) transformations of time-use composition was applied, adjusting for potential confounders. We present results for the first ilr transformations for SB, LPA, and MVPA. When analyzed the associations of bout-specific MVPA with CFD, we reworked the ilr transformations using four activities (SB, LPA, sporadic MVPA, and bouted MVPA). Model 1 was unadjusted. Model 2 was adjusted for gender and age. Model 3 was additionally adjusted for socio-demographic and behavioral factors, including residential area, educational attainment, working status, living arrangement, and BMI. Model 4 was additionally adjusted for past history of stroke and the use of medication for hypertension, dyslipidemia, and diabetes. If activity behaviors were found to be significantly associated with CFD, we estimated percent change in being CFD when fixed durations of time were reallocated from one part of a particular composition to another, while the remaining parts were kept constant.^33^^,^^34^

## RESULTS

### Participant enrollment and descriptive statistics

Of the 527 older adults who agreed to wear an accelerometer (response rate: 39.2%), 16 were excluded for: not meeting accelerometer wearing time criteria (*n* = 13), hospitalization (*n* = 2), and accelerometer system error (*n* = 1). The final analytic sample was 511 in this study.

Table 1 presents the characteristics of the participants. Overall, the mean age was 73.4 (standard deviation [SD], 5.6) years (53.0% women) and mean value of accelerometer wear time was 887.7 (SD, 108.3) min/day. Participants spent 445.6 (SD, 129.8) min/day in SB, 388.8 (SD, 103.0) min/day in LPA, 52.4 (SD, 39.9) min/day in MVPA. 48 (9.4%) older adults had CFD. Compared to NCF, those identified with CFD were significantly more likely to be older age and non-workers, and to have experienced stroke. There was no significant difference in the proportion of those adhering to global physical activity guidelines (NCF: 23.3%, CFD: 12.5%).

**Table 1.** Participant’s characteristics by cognitive status

	CFD	NCF	*P*-value
	(*n* = 48, 9.4%)	(*n* = 463, 90.6%)	
	
	*n* (%)/mean (SD)	*n* (%)/mean (SD)	
Gender, men	23 (47.9%)	217 (46.9%)	0.890^b^
Age, years	77.6 (5.4)	73.0 (5.4)	<0.001^a^
Residential area, city side	24 (50.0%)	243 (52.5%)	0.743^b^
Education, ≥13 years	7 (14.6%)	94 (20.3%)	0.344^b^
Living arrangement, with others	41 (85.4%)	425 (91.8%)	0.138^b^
Working status, working	10 (20.8%)	203 (43.8%)	0.002^b^
Body mass index, kg/m^2^	22.5 (2.9)	22.9 (3.6)	0.310^a^
Alcohol use, yes	25 (52.1%)	251 (54.2%)	0.778^b^
Smoking, yes	3 (6.3%)	41 (8.9%)	0.787^c^
Use of medication, yes			
Hypertension	28 (58.3%)	208 (44.9%)	0.076^b^
Dyslipidemia	14 (29.2%)	158 (34.1%)	0.489^b^
Diabetes	5 (10.4%)	46 (9.9%)	0.804^c^
Past history of stroke, yes	9 (18.8%)	33 (7.1%)	0.011^c^
Physical activity guidelines, meeting	6 (12.5%)	108 (23.3%)	0.086^b^
Accelerometer wear time, min/day	880.9 (159.9)	887.4 (100.5)	0.689^a^
Activity time, arithmetic mean			
SB, min/day	476.2 (153.9)	442.4 (126.8)	0.086^a^
LPA, min/day	370.9 (109.7)	390.7 (102.2)	0.205^a^
Total MVPA, min/day	33.8 (30.2)	54.3 (40.3)	<0.001^a^
sporadic MVPA, min/day	26.6 (20.5)	39.2 (25.9)	<0.001^a^
bouted MVPA, min/day	7.2 (14.3)	15.1 (22.1)	0.001^a^

CFD, cognitive function decline; LPA, light-intensity physical activity; MVPA, moderate-to-vigorous physical activity; NCF, normal cognitive function; SB, sedentary behavior; SD, standard deviation.

*P*-value was calculated using ^a^*t* test, ^b^chi-square test, or ^c^Fisher’s exact test, as appropriate.

Table 2 shows the variation matrix indicating the dispersion of each behavior. The highest log-ratio variances all involved MVPA, which indicated that time spent in MVPA was the least co-dependent on the other behaviors. The largest variability was observed in ratio of MVPA to SB.

**Table 2.** Variation matrix of time spent in activity behaviors

	SB	LPA	MVPA
SB	0		
LPA	0.273	0	
MVPA	1.299	0.789	0

LPA, light-intensity physical activity; MVPA, moderate-to-vigorous physical activity; SB, sedentary behavior.

A value close to zero implies that the times spent in the two behaviors involved in the ratio are highly proportional.

**Figure 1.**
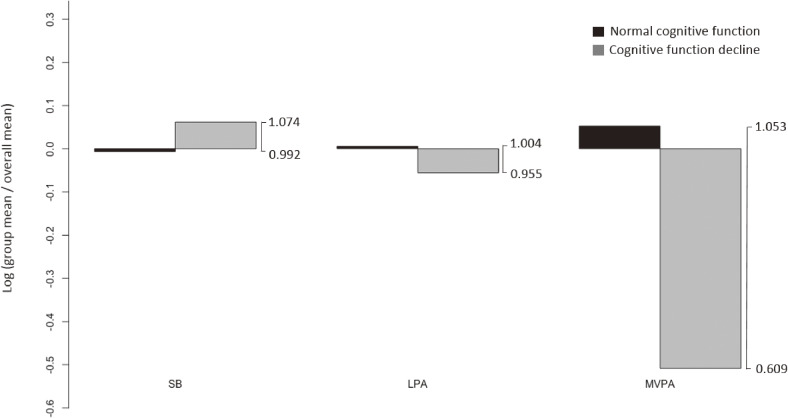
Composition of the day by cognitive status. Compositional analysis of the relative importance of the group mean time spent in SB, LPA and MVPA with respect to the overall mean time composition. In the left axis presents the log-ratio value and the right axis displays the actual proportion relative to the mean composition (eg, 1.053 means 1.053 times the compositional mean or a proportion higher by 5.3%). LPA, light-intensity physical activity; MVPA, moderate-to-vigorous physical activity; SB, sedentary behavior.

The activity composition of the day grouped by CFD status is presented in Figure 1. The MANOVA test showed a statistically significant difference in time-use activity composition between those with CFD and those with NCF. The proportion of time spent in total MVPA was reduced by 39.1% relative to the overall mean composition in CFD, while that was increased by 5.3% in NCF, the proportion of SB was higher by 7.4% and that of LPA was lower by 4.5%, relative to the mean composition. When looking at differences of bout-specific MVPA, participants with CFD had 37% less sporadic MVPA and 62% less bouted MVPA.

### Associations of sedentary and physically-active with cognitive function

Results of multiple logistic regression models are presented in Table 3. In both unadjusted and adjusted models, longer proportion of time spent in total MVPA was significantly associated with lower odds of CFD (model 4; Odds ratio [OR] 0.59; 95% confidential interval [CI], 0.36–0.94). Proportion of time spent in LPA (OR 2.19; 95% CI, 0.66–7.74) and SB (OR 1.06; 95% CI, 0.42–2.72) relative to the other behaviors were not associated with cognitive function. In bout-specific analysis, no significant associations were observed in both sporadic MVPA (OR 0.63; 95% CI, 0.31–1.26) and bouted MVPA (OR 0.93; 95% CI, 0.71–1.20).

**Table 3.** Associations of sedentary and physically-active behaviors with cognitive function in older adults

	Model 1		Model 2		Model 3		Model 4	
			
	OR	(95% CI)	OR	(95% CI)	OR	(95% CI)	OR	(95% CI)
**Activity behaviors**								
SB	1.30	(0.63, 2.70)	1.03	(0.46, 2.27)	0.90	(0.36, 2.20)	0.96	(0.38, 2.39)
LPA	1.55	(0.61, 3.92)	1.34	(0.51, 3.83)	2.04	(0.65, 6.74)	1.84	(0.58, 6.18)
MVPA	0.49	(0.33, 0.74)	0.71	(0.45, 1.12)	0.55	(0.32, 0.91)	0.57	(0.33, 0.96)
sporadic MVPA	0.64	(0.34, 1.19)	0.85	(0.44, 1.68)	0.67	(0.33, 1.36)	0.67	(0.33, 1.37)
bouted MVPA	0.87	(0.68, 1.11)	0.93	(0.72, 1.19)	0.91	(0.70, 1.18)	0.93	(0.71, 1.20)

LPA, light-intensity physical activity; MVPA, moderate-to-vigorous physical activity; OR, odds ratio; SB, sedentary behavior.

Model 1: crude model.

Model 2: adjusted for gender and age.

Model 3: adjusted for model 2+ education, body mass index, living arrangement, working status, smoking, and alcohol use.

Model 4: adjusted for model 3+ past history of stroke, and medication for hypertension, dyslipidemia, and diabetes.

Note. Isometric log-ratio (ilr) transformation was used in compositional logistic regression analyses. The odds ratio corresponds to one increase ilr coordinates.

**Figure 2.**
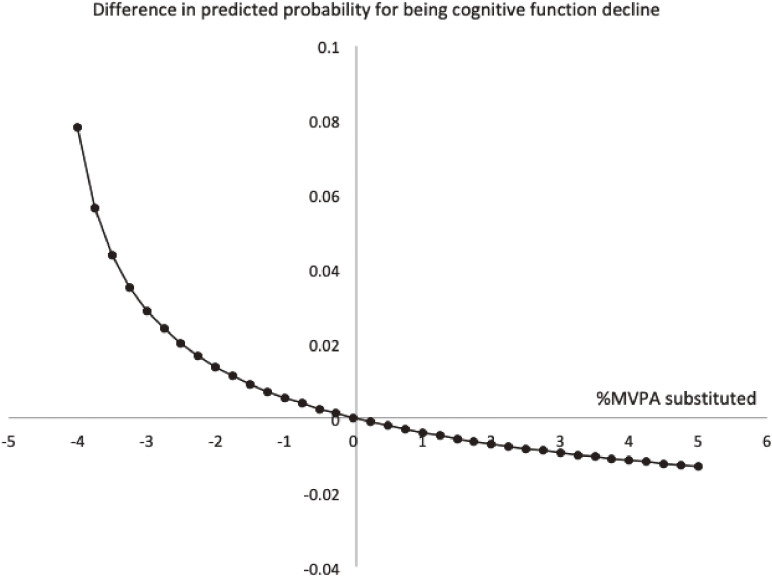
Difference in predicted probability for being cognitive function decline with reallocation of MVPA. Analyses were adjusted for gender, age, education, body mass index, living arrangement, working status, smoking, alcohol use, past history of stroke, and medication for hypertension, dyslipidemia, and diabetes. Difference in probability modelled around the population mean composition (%/day): sedentary behavior = 51.0%, light-intensity physical activity = 44.6%, moderate-to-vigorous physical activity = 4.4%. MVPA, moderate-to-vigorous physical activity.

Figure 2 shows predicted difference in CFD with reallocation of MVPA after adjustment for potential confounders. DCF was predicted to be 31.5% higher, when MVPA was lower from the mean by 2% at the expense of the remaining activities equally. If MVPA was 2% higher, DCF was predicted to be 16.1% lower.

## DISCUSSION

The proportion of time spent in MVPA relative to other behaviors (SB and LPA) was favorably associated with an index of cognitive function decline in our sample of community-dwelling older adults, even when time spent in other activity behaviors was taken into account. However, the proportion of time spent in bout-specific MVPA, LPA, and SB relative to the other behaviors were not associated with CFD when models were corrected for time spent in all activity behaviors. The current study adds novel evidence to the emerging body of research on physical activity and cognitive health using CoDa.

In this study, MVPA but not LPA had a significant association with CFD. These results are in line with previous studies indicating that intensity of physical activity may be of importance for better cognitive function.^35^^,^^36^ Physical activity intensity (peak counts), as measured by accelerometry, was found to be associated with better cognitive performance in older Australian adults.^35^ A study with older Japanese adults with MCI found device-based moderate physical activity but not LPA to be associated with hippocampal volume.^36^ Findings from randomized trials suggest that moderate-intensity exercise increases hippocampal perfusion^37^ and the size of hippocampus^38^ among healthy older adults. Although LPA makes much larger contribution to energy expenditure than MVPA in the older population,^28^ higher intensity of physical activity may be needed for maintaining cognitive health.

To date, the associations of SB with cognitive function have been inconsistent; some have suggested unfavorable associations,^9^^,^^10^ while others have suggested no associations.^11^^,^^39^ This inconsistency could be partly due to the differences of statistical approach. Most previous studies did not accurately control for time spent in other activities (ie, LPA and MVPA) when analyzing the effect of SB. Sufficient levels of MVPA may attenuate associations of SB with cognitive health.^11^ Another potential reason is that different types of SB have different impacts on cognitive function.^40^^,^^41^ A prospective cohort study with a large sample of United Kingdom adults showed television viewing and driving time to be unfavorably associated with cognitive decline, whereas non-occupational computer use was found to be favorably associated.^40^ There is also evidence that higher volumes of time spent in computer use and lower volumes of television viewing time can be related to better cognitive performance.^41^ Domain-specific SB, as distinct from overall sedentary time, should thus be considered in examining relationships with cognitive health.

### Strengths and limitations

We have reported novel findings on the relationships of older adult’s sedentary and physically-active behaviors with an indicator of CFD, through an explicit consideration of the co-dependence of time-use domains. Also, we conducted objective assessments of both sedentary and physically-active behaviors and cognitive function. Compared to self-report which involves reporting bias, device-based assessment using accelerometers can provide more accurate and reliable understanding of activity behaviors.^42^^,^^43^

The most important limitation in our study was the cross-sectional design, which does not allow us to infer any causal relationship. Longitudinal studies using CoDa approach are required to establish the links of sedentary and physically active behaviors with cognitive health. Another limitation was that the Active style Pro device cannot detect sleep, which can be associated with cognitive impairment.^44^ Although evidence of decline in cognitive function was objectively assessed using MMSE-J, which is valid and commonly used to screen dementia, further research using medical diagnose are needed to more accurately detect those who with dementia. There is also the need to consider selection bias. Accelerometry responders can be healthier and more active than non-responders,^45^ which would influence the generalizability of the present findings.

In conclusion, objectively measured time spent in MVPA, taking into account SB and LPA, was favorably associated with cognitive function in community-dwelling older adults. On the other hand, time spent in bout-specific MVPA, LPA, and SB relative to time spent in other behaviors were not associated with cognitive function when our models controlled for time spent in all activity behaviors. The shift of time from any behavior toward any form of MVPA (bouted or sporadic) is therefore likely to be beneficial for cognitive health. Our findings also suggest intensity of physical activity may be important for preventing cognitive decline. Further research using CoDa are needed to confirm our conclusions.
